# The expression level of the transcription factor Aryl hydrocarbon receptor nuclear translocator (ARNT) determines cellular survival after radiation treatment

**DOI:** 10.1186/s13014-015-0539-9

**Published:** 2015-11-16

**Authors:** Markus Mandl, Maria- Katharina Lieberum, Juergen Dunst, Reinhard Depping

**Affiliations:** Institute of Physiology, Center for Structural and Cell Biology in Medicine, University of Luebeck, Ratzeburger Allee 160, 23562 Luebeck, Germany; Klinik für Strahlentherapie, Universitaetsklinikum Schleswig-Holstein, Campus Luebeck, Ratzeburger Allee 160, 23538 Luebeck, Germany

**Keywords:** Aryl hydrocarbon receptor nuclear translocator, Clonogenic survival, HIF-1b, Hypoxia, Radioresistance, Radiotherapy

## Abstract

**Background:**

Tumour hypoxia promotes radioresistance and is associated with poor prognosis. The transcription factor Aryl hydrocarbon receptor nuclear translocator (ARNT), also designated as Hypoxia-inducible factor (HIF)-1β, is part of the HIF pathway which mediates cellular adaptations to oxygen deprivation and facilitates tumour progression.

The subunits HIF-1α and ARNT are key players within this pathway. HIF-1α is regulated in an oxygen-dependent manner whereas ARNT is considered to be constitutively expressed. However, there is mounting evidence that certain tumour cells are capable to elevate ARNT in hypoxia which suggests a survival benefit.

Therefore the objective of this study was to elucidate effects of an altered ARNT expression level on the cellular response to radiation.

**Methods:**

Different human cell lines (Hep3B, MCF-7, 786-Owt, 786-Ovhl, RCC4wt and RCC4vhl) originating from various tumour entities (Hepatocellular carcinoma, breast cancer and renal cell carcinoma respectively) were X-irradiated using a conventional linear accelerator. Knockdown of ARNT expression was achieved by transient siRNA transfection. Complementary experiments were performed by forced ARNT overexpression using appropriate plasmids. Presence/absence of ARNT protein was confirmed by Western blot analysis. Clonogenic survival assays were performed in order to determine cellular survival post irradiation. Statistical comparison of two groups was achieved by the unpaired *t*-test.

**Results:**

The results of this study indicate that ARNT depletion renders tumour cells susceptible to radiation whereas overexpression of this transcription factor confers radioresistance.

**Conclusions:**

These findings provide evidence to consider ARNT as a drug target and as a predictive marker in clinical applications concerning the response to radiation.

**Electronic supplementary material:**

The online version of this article (doi:10.1186/s13014-015-0539-9) contains supplementary material, which is available to authorized users.

## Background

Treatment of cancer by radiation is a common modality in oncology and applied depending on tumour entity and staging [1–4]. In breast cancer patients, adjuvant radiotherapy reduces the risk of recurrence and improves overall survival [3]. In radioresistant malignancies such as hepatocellular carcinoma (HCC) and renal cell carcinoma (RCC) radiotherapy is primarily used in a palliative setting in order to control metastatic spread [4, 5].

The resistance/sensitivity of tumour cells to radiation is governed by the intracellular oxygen (O_2_) concentration [6]. Oxygen is a biologically important element required as electron acceptor in mitochondrial energy generation [7, 8]. Additionally, it is a potent radiosensitizer due to its chemical properties as a highly reactive electrophile [6]. The oxygenation of solid tumours is heterogeneous and depends on the distance to the nearest blood vessel [9]. Oxygen deprived (i.e., hypoxic) areas are commonly found within neoplasms due to an excessive cell proliferation and are associated with increased radioresistance [1, 10, 11]. In general, tumour hypoxia is considered as a prognostic parameter predicting poor overall- and disease-free survival independent of the tumour grade [6].

In order to survive, tumour cells are forced to adapt to hypoxic conditions [7, 12]. This is mainly mediated by activation of the Hypoxia-inducible factor (HIF) pathway which consists of several transcription factors of the Per-ARNT-Sim family [12]. HIF target genes (e.g., *Vascular endothelial growth factor (VEGF)*, *Lactate dehydrogenase (LDH)*, etc.) regulate a number of cellular responses including metabolic alterations leading to reduced mitochondrial oxygen consumption and augmented glycolysis [12, 13]. The induction of angiogenesis and invasion/metastasis are HIF-dependent processes accelerating tumour progression. Furthermore, HIF signalling contributes to the cancer stem-cell phenotype and radioresistance [12, 14, 15]. Therefore inhibition of the HIF pathway in various ways is considered as a treatment option in cancer therapy [2, 16–21] and as an opportunity to overcome radioresistance [1].

Three structurally related HIF-α subunits have been described which are regulated in a similar manner but differ in their pattern of expression. HIF-1α is ubiquitously expressed whereas HIF-2α is more restricted to certain cell types [12, 22]. HIF-3α exists in several splice variants which can activate and inhibit hypoxia-dependent gene expression [23]. The impact of specific HIF-α subunits on tumourigenesis relies on the cellular context [24]. For instance, both HIF-1α and HIF-2α are correlated with poor prognosis in breast cancer [24] whereas clear cell renal carcinomas are addicted to HIF-2α [22].

The subunit HIF-1α plays a key role in the HIF pathway and is regulated in an oxygen-dependent manner. Under normoxic conditions HIF-1α is hydroxylated at two conserved proline residues by specific Prolylhydroxylase domain (PHD) enzymes. Subsequently this post-translational modification is recognized by the von Hippel-Lindau tumour suppressor protein (pVHL) which is part of an ubiquitin ligase complex and mediates the proteasomal degradation of HIF-1α [12, 14, 15]. In contrast, oxygen deprivation prevents enzymatic activity of PHDs thus leading to accumulation of HIF-1α and nuclear translocation [25]. Within the nucleus, HIF-1α binds to the Aryl hydrocarbon receptor nuclear translocator (ARNT), also known as HIF-1β, and forms the transcriptional active HIF-1 complex [14]. Afterwards target gene expression is initiated by binding of HIF-1 to hypoxia-responsive elements within the promoter sequence in conjunction with co-factors such as CBP/p300 [12, 14, 15].

An equivalent mechanism applies for HIF-2α and specific HIF-3α variants. In general, all HIF-α subunits are capable to heterodimerize with ARNT in order to form functional HIF complexes (HIF-1, HIF-2 and HIF-3 respectively) [12, 23]. Therefore a competition of HIF-α subunits in binding to ARNT was proposed [23].

In contrast, ARNT is generally regarded to be constitutively expressed meaning to be unaffected by oxygen tension. However, there is mounting evidence that certain cell types are capable to elevate ARNT in response to hypoxia [15, 26, 27]. It was demonstrated, that hypoxia-dependent upregulation of ARNT was mediated in a HIF-1α-dependent manner in human melanoma cells [26].

Therefore it is reasonable to hypothesize that an elevated ARNT expression level might provide a clonal advantage for tumour cells. The aim of this study was to elucidate the effects of ARNT silencing and overexpression in different tumour cell lines regarding radioresistance.

## Methods

### Cell culture

The human breast adenocarcinoma cell line MCF-7 (ATCC) and human renal carcinoma cells 786-Owt, 786-Ovhl, RCC4wt and RCC4vhl (all described in [28]) were maintained in DMEM high glucose medium (Gibco®) supplemented with 10 % fetal bovine serum (FBS, Gibco®) and Penicillin/Streptomycin. Human hepatocellular carcinoma Hep3B cells (ATCC) were cultured in RPMI 1640 medium (Gibco®) supplemented with 10 % FBS and Penicillin/Streptomycin. Cell cultures were maintained at 37 °C in a humidified atmosphere containing 5 % v/v CO_2_. Cells were harvested by trypsinization and subcultured at least twice a week in a ratio of 1:5 – 1:10.

### Hypoxic exposure

1,7 × 10^6^ cells were plated on 10 cm Petri-dishes and allowed to adhere overnight. On the next day, the supernatant was replaced by 10 ml fresh growth medium and cells were exposed to hypoxia (3 % v/v O_2_) for 8 h at 37 °C using a standard cell culture incubator with saturated humidified atmosphere (5 % v/v CO_2_, balanced N_2_). For control, cells were maintained under normoxic standard cell culture conditions.

### siRNA transfection

Gene silencing was achieved by a reverse transfection procedure using Lipofectamine® RNAiMAX (Life technologies) in accordance with the manufacturer’s guidelines. Therefore, cells were seeded on 24-well plates and mixed with the transfection mixture containing either ARNT siRNA (#s1613, Ambion) or BLOCK-iT™ Fluorescent Control siRNA (#442926, Life technologies). Subsequently cells were incubated overnight and subjected to irradiation. To gain protein lysates for Western blot analysis, the transfection procedure was performed in 6-well plates.

### Transient plasmid transfection

4 × 10^4^ cells/well were seeded in 24-well plates using antibiotic-free growth medium and incubated overnight. On the next day, the medium was renewed and transfection procedure was performed using GeneJuice® (Merck Millipore) according to the supplier’s protocol. Cells were transfected overnight either with 0.5 μg DNA/well of an appropriate ARNT expression vector (pcDNA3-ARNT) or the empty plasmid for control (pcDNA3). Subsequently cells were subjected to irradiation. To gain protein lysates for Western Blot analysis, transfection procedure was up-scaled and performed in 6-well plates.

### Irradiation of cells and clonogenic survival assays

Untransfected cells were seeded in 24-well plates at a density of 5 × 10^4^ cells/well 1 day before irradiation. X-rays were applied using a conventional linear accelerator (Mevatron-74, Siemens or Clinac DHX, Varian) at doses ranging from 0 to 10 Gray (Gy) with 2Gy intervals. Clonogenic survival of cells was assayed as described previously [2]. Briefly, cells were harvested after irradiation using Accutase™ (PAA), counted by an automated trypan blue-exclusion technique (Cellometer™, Nexcelom Bioscience) and seeded in triplicates in 6-well plates at a defined density. Subsequently cells were grown in normoxia for 9 or 12 days (MCF-7). Finally, cells were washed with cold Phosphate buffered saline (PBS), fixed with 3.7 % Formaldehyde and 70 % Ethanol. Colonies were stained with Coomassie and counted. Clonogenic survival of irradiated cells was calculated on a percentage basis compared to appropriate non-irradiated controls.

### Gene expression analysis

Gene expression was analysed by quantitative reverse transcription – polymerase chain reaction (qRT-PCR). Therefore total RNA was isolated using the innuPREP RNA Mini Kit (Analytik Jena) as described in the supplier’s protocol. Reverse transcription was performed using oligo-dT primer and M-MuLV Reverse Transcriptase (New England Biolabs) in accordance with the manufacturer’s guidelines. ARNT mRNA expression was measured using TaqMan® Gene Expression Assays (#Hs01121918_m1, Applied Biosystems) and compared to endogenous Beta-2-microglobulin (B2M) mRNA expression levels (#Hs00984230_m1, TaqMan® Gene Expression Assays, Applied Biosystems). Quantitative real-time PCR was carried out on an ABI PRISM® 7000 instrument (Applied Biosystems) applying the protocol for comparative relative quantitation (∆∆C_t_ method).

### Western blot analysis

Cells were lysed using urea buffer as described previously [2, 27]. The protein concentration of sonicated cell lysates was determined using the DC™ Protein Assay (Bio-Rad) according to the supplier’s guidelines. A total protein amount of 50 μg/lane was dissolved on a 7.5 % acrylamide gel and blotted onto Polyvinyl difluoride (PVDF) membrane (Immobilon-P, 0.45 μm, Merck Millipore) using the semi-dry technique. Unspecific binding sites were blocked with 5 % non-fat dry milk/PBS for 1 h. Membranes were probed with anti-HIF-1α (1:1000, clone 54/HIF-1α, #610959, BD Transduction Laboratories™), anti-HIF-2α (1:1000, polyclonal, #NB100-122, Novus Biologicals) or anti-ARNT (1:2000, clone 2B10, #NB300-525, Novus Biologicals) antibodies overnight agitating at 4 °C. Equal loading of samples and transfer was verified using an anti-Actin (1:2000, polyclonal, #sc-1615, Santa Cruz Biotechnology) antibody applied for 1 h at room temperature. Appropriate HRP-conjugated secondary antibodies (DAKO, 1:5000) were applied for 1 h at room temperature followed by chemo-luminescence detection using the ECL reagent (Clarity™ Western ECL, Bio Rad). Finally membranes were exposed to X-ray films (Amersham Hyperfilm™ MP, GE Healthcare).

### Statistics

Statistical analysis was performed using GraphPad Prism® 4 software (GraphPad). All values are presented as mean +/− standard error of the mean (SEM). Each experiment was repeated at least three times. Clonogenic survival curves represent the mean +/− SEM of at least five independent experiments. The unpaired *t*-test was applied to compare two groups. *P* values ≤0.05 were considered as statistically significant.

## Results

### ARNT depletion renders tumour cells susceptible to radiation

**Fig. 1:**
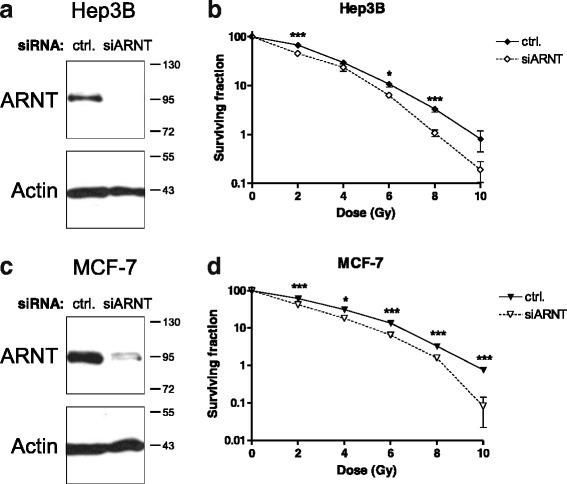
Effect of ARNT knockdown on radioresistance. **a** Hep3B cells were transfected with non-target control siRNA (ctrl.) or siRNA against ARNT (siARNT) and subjected to Western blot analysis. Actin levels were determined for loading control. Protein masses are indicated on the right in kDa. **b** Clonogenic survival assays of control- (ctrl.) or ARNT siRNA (siARNT) transfected and irradiated Hep3B cells. *n* = 6, mean +/− SEM, unpaired *t*-test; (**c**) MCF-7 cells were transfected and Western blot analysis performed as described in a). **d** Clonogenic survival assays of irradiated MCF-7cells transfected as described in (**b**). *n* = 6, mean +/− SEM, unpaired *t*-test

Human Hep3B cells are capable to upregulate ARNT in response to reduced oxygen supply as demonstrated by previous studies [27, 29]. In order to investigate the importance of this transcription factor in cellular radioresistance, ARNT was knocked down in Hep3B cells using siRNA. Western blot analysis confirmed the successful depletion of the transcription factor in ARNT-siRNA transfected Hep3B cells (Fig. 1a).

The same siRNA-based approach was applied prior radiation treatment. Therefore Hep3B cells were again transfected either with a non-target control or ARNT siRNA and irradiated with doses from 2 to 10 Gy. Subsequently the tumour-initiating capacity of cells was measured by clonogenic survival assays. As shown in Fig. 1b (and Additional file 1: Figure S1a), silencing of ARNT significantly reduces radioresistance in Hep3B cells compared to appropriate controls.

In order to test whether ARNT influences the response of human breast cancer cells to radiation, MCF-7 cells, which represent one of the most widely used models of this malignancy [30], were transfected either with control- or ARNT siRNA. The functionality of the knockdown procedure was tested by Western blotting (Fig. 1c). Irradiated ARNT-silenced MCF-7 cells exhibited a significant decrease in radioresistance as compared to control cells (Fig. 1d, Additional file 1: Figure S1b).

These findings indicate a key role of the transcription factor ARNT mediating cellular radioresistance.

### ARNT mRNA and protein expression differs among renal cell carcinoma cell lines and depends on pVHL status

**Fig. 2:**
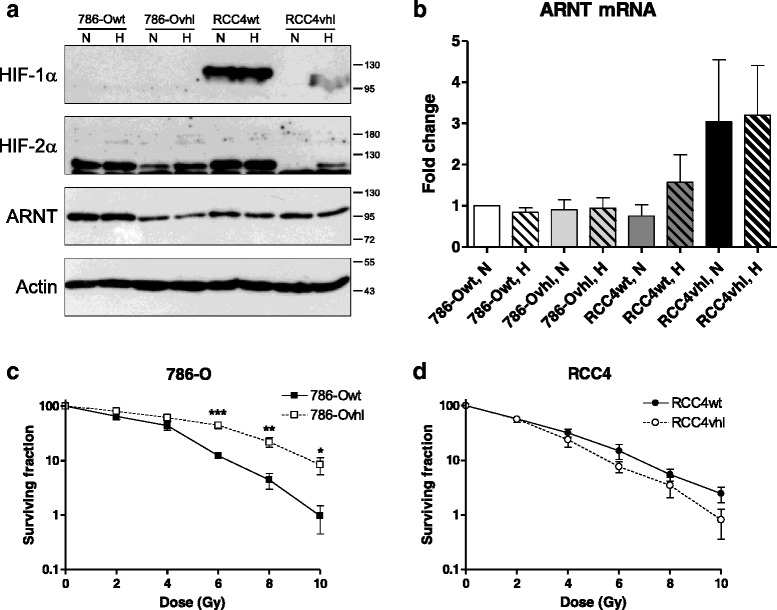
ARNT expression in renal carcinoma cells and response to radiation. **a** 786-O and RCC4 wildtype (wt) and pVHL expressing (vhl) cells respectively were seeded on Petri-dishes followed by exposure to normoxia (N) or hypoxia (H, 3 % O_2_) for 8 h. Protein levels of HIF-1α, HIF-2α and ARNT were assayed by Western Blotting. Actin levels were determined for loading control. Protein masses are indicated on the right in kDa. Representative result of *n* = 3 independent experiments. **b** ARNT mRNA expression in 786-Owt, 786-Ovhl, RCC4wt and RCC4vhl cells exposed to normoxia (N) or hypoxia (H, 3 % O_2_) for 8 h. ARNT- and B2M (endogenous control) mRNA were measured using TaqMan® chemistry and normalized to normoxic 786-Owt cells according to the ∆∆C_t_ method. Fold changes of ARNT mRNA levels are represented as mean +/− SEM of *n* = 3 independent experiments. **c** Clonogenic survival assays of irradiated 786-Owt and 786-Ovhl cells. *n* = 6, mean +/− SEM, unpaired *t*-test; (**d**) Clonogenic survival assays of irradiated RCC4wt and RCC4vhl cells. *n* = 5–6, mean +/− SEM, unpaired *t*-test

Renal carcinoma cells are characterized by constitutive HIF signalling due to pVHL loss of function [22]. In order to test whether ARNT is affected by pVHL-status in addition to reduced oxygen supply in this cell type, 786-O and RCC4 wildtype (wt) and stably pVHL-transfected counterparts (vhl) were subjected to 3 % O_2_ for 8 h or maintained in normoxia for control. As shown in Fig. 2a, 786-O cells lack HIF-1α. As expected, HIF-2α protein was pronounced expressed even under normoxic conditions in 786-Owt cells. In contrast, HIF-2α was detected in normoxic 786-Ovhl cells at lower levels compared to wildtype counterparts. Elevation of HIF-2α due to hypoxic exposure demonstrates a functional canonical HIF pathway in 786-Ovhl cells. ARNT protein levels were unaffected by oxygen deprivation in both 786-Owt and 786-Ovhl cells. Interestingly, ARNT was decreased in stably transfected 786-Ovhl cells compared to wildtype controls under normoxic and hypoxic conditions.

HIF-1α and HIF-2α were profoundly expressed in RCC4wt cells independent of oxygen tension. Both subunits were not detected in normoxic RCC4vhl cells but accumulate under hypoxic conditions which indicate canonical HIF signalling. ARNT protein level was equal in RCC4wt and RCC4vhl cells and unaffected by hypoxia.

To confirm these findings, ARNT mRNA levels were measured by qRT-PCR using the same experimental conditions and normalized to normoxic 786-Owt cells (Fig. 2b). Noteworthy and in contrast to ARNT protein level, ARNT mRNA expression was equal in both 786-Owt and 786-Ovhl cells independent of oxygen tension.

In order to test whether pVHL expression facilitates proteasomal degradation of ARNT, 786-Ovhl cells were treated with various concentrations of the proteasome inhibitor MG-132. ARNT protein expression was not rescued by MG-132 treatment (not shown) in 786-Ovhl cells thus indicating a post-transcriptional mechanism.

The ARNT mRNA level in RCC4wt cells was slightly elevated in hypoxia compared to normoxic controls (Fig. 2b). Stably pVHL expressing RCC4vhl counterparts exhibited an approximately three fold increase of ARNT mRNA independent of oxygen tension. Interestingly, this effect was not accompanied with an appropriate increase of ARNT protein level in RCC4vhl cells (Fig. 2a). These findings again point towards a pVHL-dependent regulation on ARNT mRNA level in this model system.

### Radiosensitivity of renal carcinoma cells is influenced by pVHL

Next, radiosensitivity of renal carcinoma cell lines was investigated. As shown in Fig. 2c (and Additional file 2: Figure S2a), 786-Ovhl cells were significantly more resistant to radiation treatment compared to wildtype counterparts. Interestingly, an opposite effect was observed in the RCC4 model system (Fig. 2d, Additional file 2: Figure S2b). Herein stably transfected RCC4vhl cells were more sensitive to X-rays as compared to RCC4wt cells. These results demonstrate that pVHL affects radiosensitivity in a cell-specific manner.

### Effects of ARNT on radiosensitivity of 786-Owt cells

**Fig. 3:**
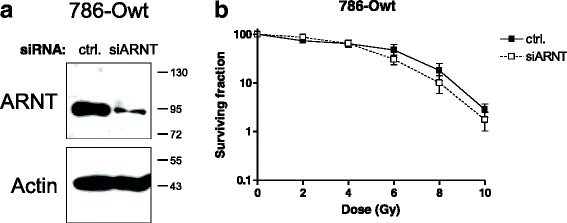
Knockdown of ARNT in 786-Owt cells and effects on radioresistance. **a** 786-Owt cells were transfected with non-target control (ctrl.) siRNA or siRNA against ARNT (siARNT) and subjected to Western blot analysis. Actin levels were determined for loading control. Protein masses are indicated on the right in kDa. **b** Clonogenic survival assays of irradiated 786-Owt cells transfected as described in (**a**). *n* = 6, mean +/− SEM, unpaired *t*-test

786-Owt cells exhibited an elevated ARNT protein level compared to stably transfected 786-Ovhl cells (Fig. 2a). In order to test whether ARNT affects radiosensitivity in this cell line, the transcription factor was silenced by siRNA prior to irradiation. Figure 3a shows the successful depletion of the transcription factor in ARNT-siRNA transfected 786-Owt cells. As shown in Fig. 3b, silencing of ARNT rendered 786-Owt cells more susceptible to radiation beyond doses of 4Gy as compared to appropriate control cells.

### Overexpression of ARNT confers tumour cells a radioresistant phenotype

**Fig. 4:**
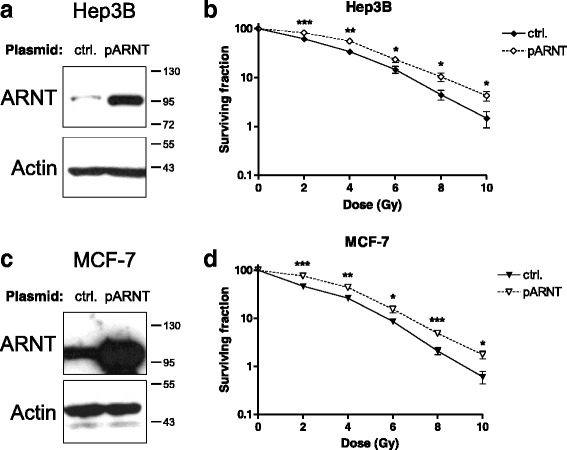
Effects of elevated ARNT expression on radioresistance. **a** Hep3B cells were transfected either with the empty plasmid for control (ctrl.) or an appropriate ARNT expression vector (pARNT) and subjected to Western blot analysis. Actin levels were determined to ensure equal loading of samples. Protein masses are indicated on the right in kDa. **b** Clonogenic survival assays of irradiated Hep3B cells transfected as described in (**a**). *n* = 6, mean +/− SEM, unpaired *t*-test; (**c**) MCF-7 cells were transfected as indicated in (**a**) and subjected to Western Blotting. Actin levels were determined to ensure equal loading of samples. Protein masses are given in kDa. **d** Clonogenic survival assays of irradiated MCF-7 cells transfected as described in (**a**). *n* = 6, mean +/− SEM, unpaired *t*-test

In order to test whether an increased ARNT expression might affect radiosensitivity, Hep3B cells were transiently transfected either with an appropriate expression- or empty control vector prior to irradiation. Elevated ARNT protein expression in appropriate transfected cells was analysed as shown in Fig. 4a. Clonogenic survival assays of irradiated ARNT-overexpressing Hep3B cells revealed a significant increase in radioresistance compared to vector-transfected controls (Fig. 4b, Additional file 3: Figure S3a).

To investigate whether ARNT overexpression also shifts breast cancer cells towards a more radioresistant phenotype, MCF-7 cells were transiently transfected as described above. Successful transfection and ARNT overexpression in this cell line was confirmed as shown in Fig. 4c. ARNT-overexpressing MCF-7 cells exhibited an increased resistance to radiation as compared to appropriate control cells (Fig. 4d, Additional file 3: Figure S3b).

The results of these complementary experiments indicate a clonal benefit of an elevated ARNT expression in tumour cells regarding the cellular survival after radiation treatment.

## Discussion

The results of this study demonstrate a novel and more pronounced role of the transcription factor ARNT regarding the cellular survival after irradiation. Depletion of ARNT by siRNA conferred Hep3B and MCF-7 tumour cells a radiosensitive phenotype whereas overexpression promotes radioresistance. This quantitative effect indicates that an elevated ARNT expression provides a selective survival benefit after radiation treatment in these cell models. Therefore it is reasonable to hypothesize whether radiotherapy might be a selection pressure and facilitates the enrichment of high ARNT expressing cells in hepatocellular carcinoma and breast cancer respectively. This issue should be addressed in future studies. For instance, by analysis of appropriate clinical samples using immunohistochemistry.

A similar radio-sensitizing effect was observed in ARNT-silenced 786-Owt renal carcinoma cells at high doses. Interestingly, the results demonstrate that pVHL status affects radiosensitivity and ARNT mRNA and protein expression in both renal carcinoma cell models analysed. These effects are cell-context dependent and differ among 786-O and RCC4 cells. This raises the question regarding the mechanistic linkage between pVHL and ARNT expression.

It was demonstrated that kidney cancers exhibit increased NF-κB activity and that pVHL is a negative regulator of this pathway [31]. In addition, *Van Uden* et al. showed that ARNT is a NF-κB target gene [32]. A similar mechanism might therefore explain the reduced ARNT protein level in 786-Ovhl cells.

Furthermore, it was reported that pVHL is a multifunctional protein also influencing the mRNA stability of certain genes. In 786-O cells, pVHL affects the expression of approximately 800 genes which strongly suggests a more complex role beyond its participation in the HIF pathway [33]. However, the exact mechanism how pVHL regulates ARNT expression remains to be elucidated.

Renal cancer is a radioresistant malignancy [4] and efforts have been made in order to improve radiosensitivity [34]. For instance, treatment of renal carcinoma cells with the anti-inflammatory drug Ibuprofen resulted only in moderate effects [34]. Targeting the pVHL/HIF pathway in an effective way was proposed as a treatment option in renal cell carcinoma [35] but quantitative and qualitative differences among HIF-α subunits in this malignancy need to be considered [22]. In general, HIF inhibition is regarded to be beneficial in anti-cancer therapy [2, 16, 36].

ARNT was also anticipated as a profound therapeutic target in certain types of cancer [37]. Indeed, the data presented in our study supports this notion. Inhibition of ARNT expression might therefore render appropriate tumour cells more susceptible to radiotherapy.

According to the majority of literature, ARNT is regarded to be constitutively expressed but certain tumour cell lines are capable to elevate ARNT in hypoxia [15, 26, 27]. Investigating the molecular mechanism of ARNT expression under oxygen deprivation might reveal further opportunities for intervention in order to promote radiosensitivity.

The “drugability” of a target is important for validation and clinical application. One option is the prevention of protein-protein interactions which are mediated via large domains [38]. The PAS domains are conserved structures among HIF proteins including ARNT [12]. Studies have demonstrated that blocking of PAS domains by specific inhibitors is a feasible approach to prevent heterodimerization among subunits [18, 39, 40]. Recently, an ARNT inhibitor was described by *Guo* et al. which selectively interacts with the PAS-B domain [17]. It is likely that the described pro-survival effects of ARNT in our study are mediated by interaction with a HIF-α subunit.

*Isaacs* et al. demonstrated, that ARNT can stabilize its binding partner HIF-1α [41]. Thus an elevated ARNT level might lead to a prolonged HIF signalling after irradiation. Avoidance of HIF-α/ARNT heterodimerization by an appropriate inhibitor or small molecule ligand might therefore contribute to radiosensitivity.

Another possible mechanism to prevent ARNT from exerting its function as a transcription factor might be to modulate nuclear translocation. It was demonstrated that HIFs are transferred into the nucleus in an importin-dependent manner [42]. Additionally, it was proposed that targeted mislocalization of proteins might be a promising strategy in cancer therapy [14].

Moreover, *Harada* et al. showed that cancer cells acquire HIF-1 activity after radiotherapy [43]. Our results are in-line with this observation demonstrating that ARNT is required for clonogenic cell survival after irradiation. The fact that ARNT overexpression promotes a radioresistant phenotype in Hep3B and MCF-7 cells supports the concept of ARNT as being a limiting factor in HIF signalling as previously proposed [26].

## Conclusions

The transcription factor Aryl hydrocarbon receptor nuclear translocator (ARNT), also known as Hypoxia-inducible factor (HIF)-1β, is part of the HIF signalling pathway which mediates cellular adaptations to oxygen deprivation and contributes to radioresistance of neoplasms. ARNT is generally considered as constitutively expressed but emerging evidence indicates the capability of certain tumour cells to upregulate ARNT in response to hypoxia. Thus an elevated ARNT expression level might provide a clonal advantage for tumour cells.

The results of our study demonstrate that ARNT depletion renders tumour cells susceptible to radiation whereas overexpression of this transcription factor promotes radioresistance. These findings provide a rationale to consider ARNT as a drug target and as a predictive marker in clinical applications regarding the response to radiation.

## Additional files

Additional file 1: Figure S1. Clonogenic survival assays of irradiated siRNA-transfected cells. a) Hep3B cells b) MCF-7 cells; ctrl: control-siRNA transfected; siARNT: silenced ARNT; Representative result of *n* = 6 independent experiments. (PPTX 2478 kb) Additional file 2: Figure S2. Clonogenic survival assays of irradiated renal cell carcinoma cells. a) 786-Owt vs. 786-Ovhl. b) RCC4wt vs. RCC4vhl. Representative result of *n* = 5–6 independent experiments. (PPTX 4610 kb) Additional file 3: Figure S3. Clonogenic survival assays of irradiated plasmid-transfected cells. a) Hep3B cells. b) MCF-7 cells; Representative result of *n* = 6 independent experiments. ctrl: control plasmid transfected cells; pARNT: ARNT expression vector transfected. (PPTX 19264 kb)
