# Supplementary figures and images for: The expression level of the transcription factor Aryl hydrocarbon receptor nuclear translocator (ARNT) determines cellular survival after radiation treatment
Source: Radiat Oncol. 2015 Nov 16;10:229. doi: 10.1186/s13014-015-0539-9 (PMC4647475; doi:10.1186/s13014-015-0539-9)

## Slide 1
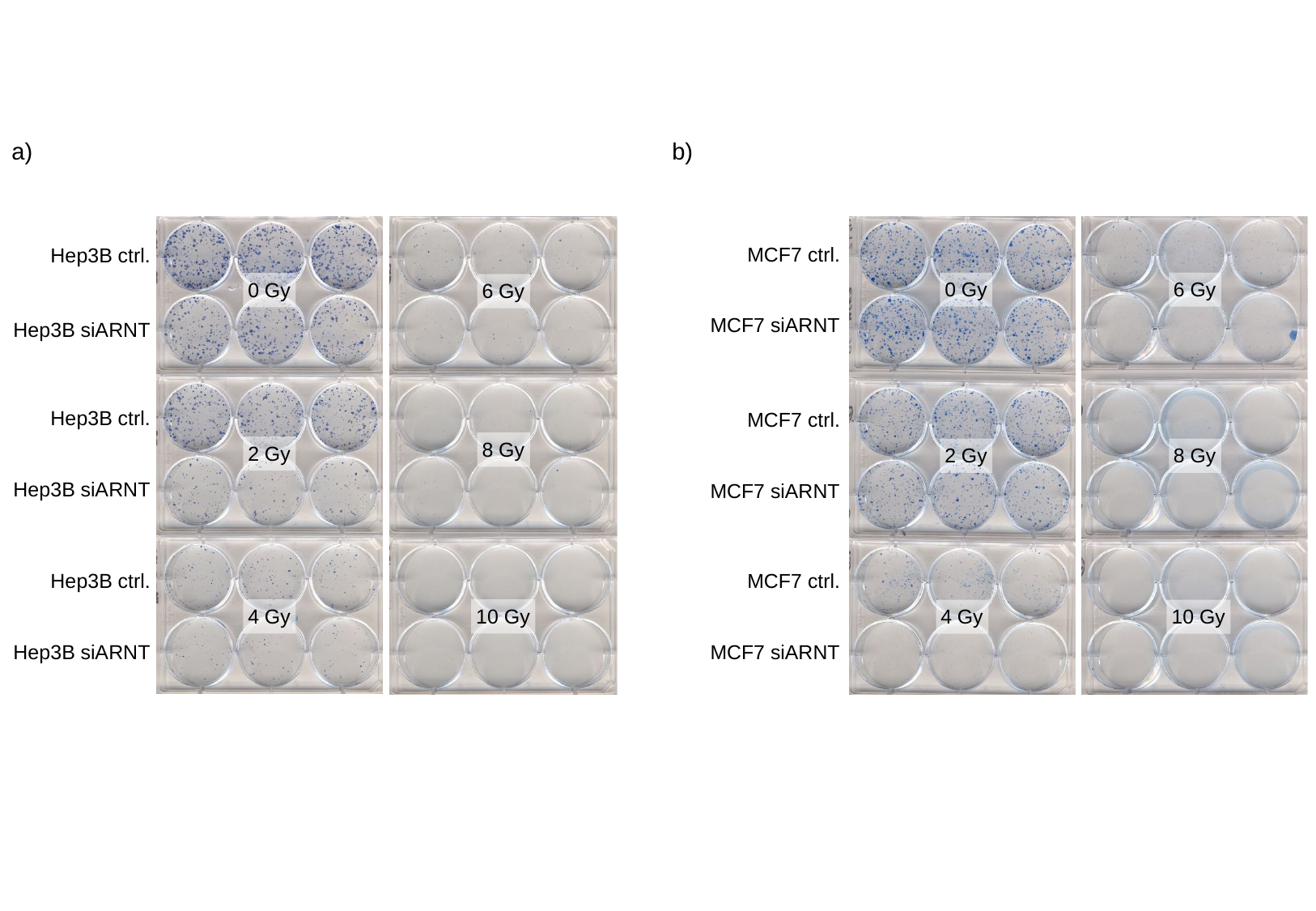

a)					 b)

Supplement: Additional file 1: Figure S1. — Clonogenic survival assays of irradiated siRNA-transfected cells. a) Hep3B cells b) MCF-7 cells; ctrl: control-siRNA transfected; siARNT: silenced ARNT; Representative result of n = 6 independent experiments. (PPTX 2478 kb) [file 13014_2015_539_MOESM1_ESM.pptx]

## Slide 1
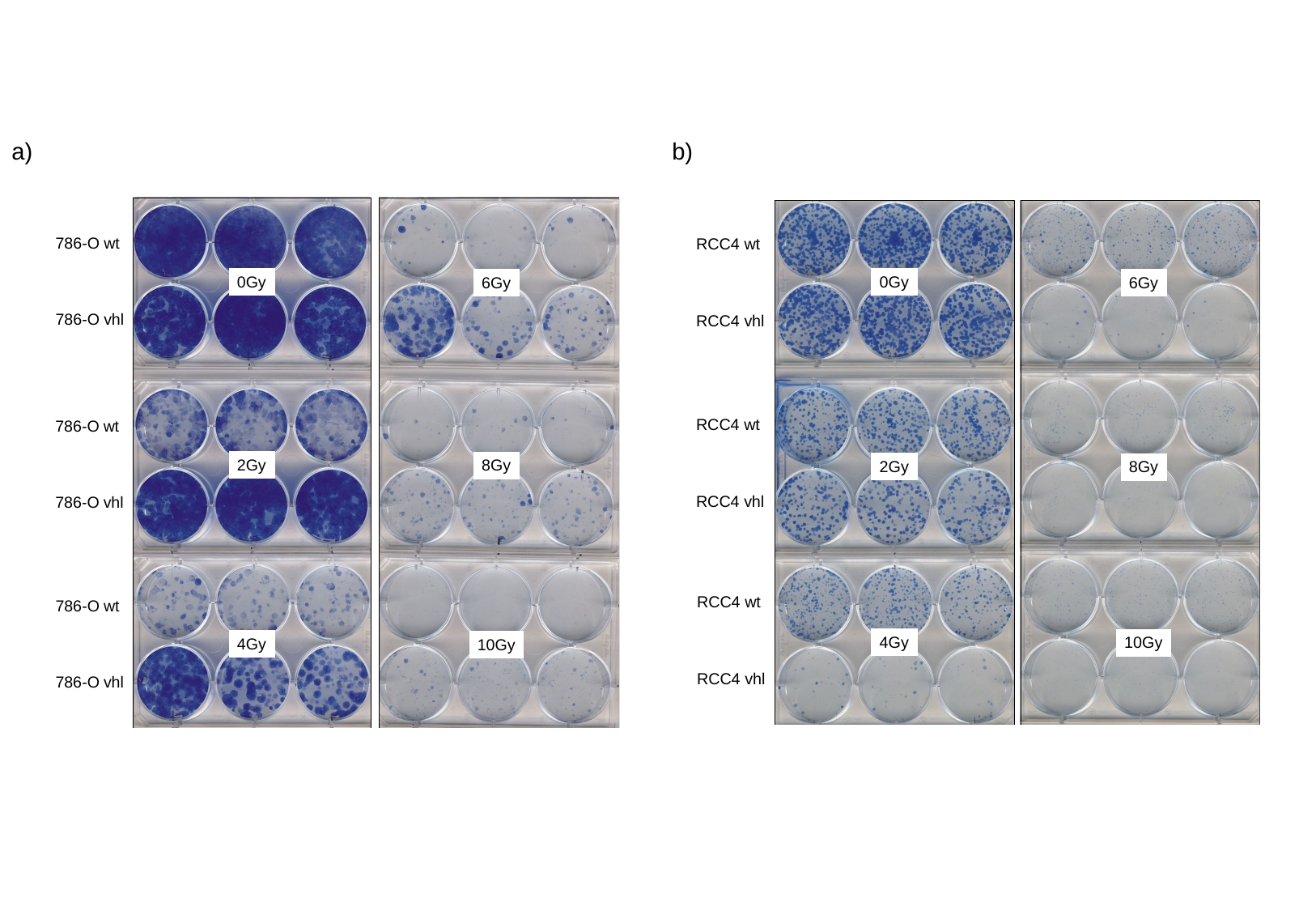

a)					 b)

Supplement: Additional file 2: Figure S2. — Clonogenic survival assays of irradiated renal cell carcinoma cells. a) 786-Owt vs. 786-Ovhl. b) RCC4wt vs. RCC4vhl. Representative result of n = 5–6 independent experiments. (PPTX 4610 kb) [file 13014_2015_539_MOESM2_ESM.pptx]

## Slide 1
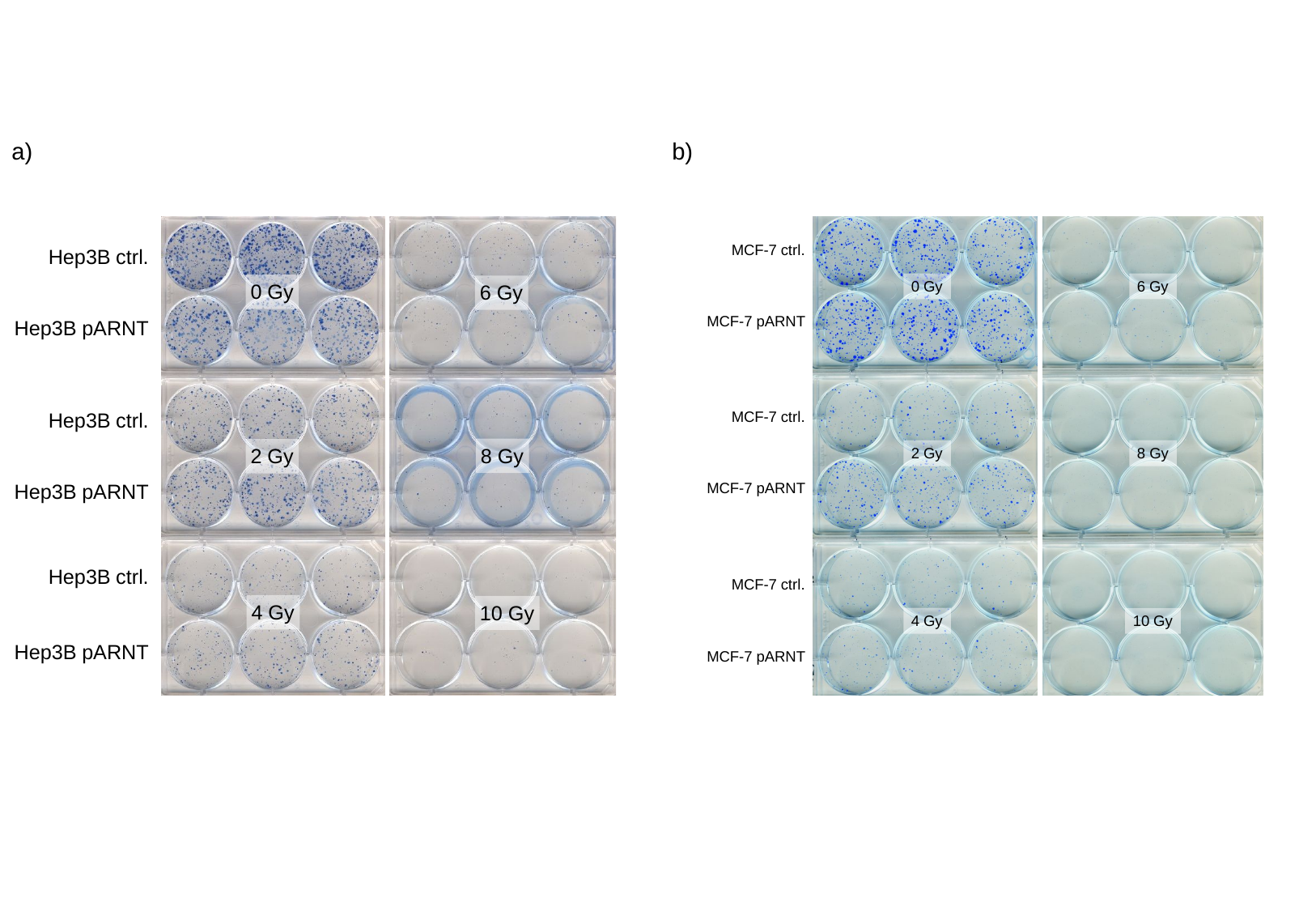

a)					 b)

Supplement: Additional file 3: Figure S3. — Clonogenic survival assays of irradiated plasmid-transfected cells. a) Hep3B cells. b) MCF-7 cells; Representative result of n = 6 independent experiments. ctrl: control plasmid transfected cells; pARNT: ARNT expression vector transfected. (PPTX 19264 kb) [file 13014_2015_539_MOESM3_ESM.pptx]
